# Household Pasteurization of Drinking-water: The *Chulli* Water-treatment System

**Published:** 2006-09

**Authors:** Mohammad Fakhrul Islam, Richard B. Johnston

**Affiliations:** ^1^ Applied Chemistry and Chemical Technology, Rajshahi University, Rajshahi, Bangladesh; ^2^ Water and Environmental Sanitation Section, United Nations Children's Fund, BSL Office Complex, 1 Minto Road, Dhaka 1000, Bangladesh

**Keywords:** Drinking-water, Arsenic, Arsenic contamination, Water treatment, Water quality, Pasteurization, Bacteria, Disinfection, Water supply, Thermal inactivation, Bangladesh

## Abstract

A simple flow-through system has been developed which makes use of wasted heat generated in traditional clay ovens (*chullis*) to pasteurize surface water. A hollow aluminium coil is built into the clay *chulli*, and water is passed through the coil during normal cooking events. By adjusting the flow rate, effluent temperature can be maintained at approximately 70 °C. Laboratory testing, along with over 400 field tests on *chulli* systems deployed in six pilot villages, showed that the treatment completely inactivated thermotolerant coliforms. The *chulli* system produces up to 90 litres per day of treated water at the household level, without any additional time or fuel requirement. The technology has been developed to provide a safe alternative source of drinking-water in arsenic-contaminated areas, but can also have wide application wherever people consume microbiologically-contaminated water.

## INTRODUCTION

Since the discovery of substantial arsenic contamination of groundwater in Bangladesh, a number of alternatives to shallow groundwater have been promoted to supply drinking-water in arsenic-affected areas. These include deep tubewells, slow sand filters (called pond-sand filters), dugwells, rooftop rainwater harvesting, and arsenic-removal systems. Each method has its own merits and demerits and may be more or less appropriate in different regions of the country.

Much effort has been devoted to finding ways to make safe use of surface water for drinking and cooking, as it is abundant in many parts of the country for much of the year. Furthermore, the National Policy for Arsenic Mitigation states that surface water should be given preference to groundwater for mitigation of arsenic, partly because of concerns about eventual contamination of currently arsenic-free aquifers (1). Surface water is generally free from arsenic in Bangladesh. However, as surface water is often highly turbid and usually highly contaminated with faecal material, steps must be taken to improve the physical and microbiological quality before consumption. Suspended solids are easily removed through filtration but microbiological contamination presents more of a challenge. Rapid filtration may remove protozoa and cysts, but bacteria and viruses can pass through simple sand filters. In principle, slow sand filtration can provide excellent removal of protozoa, bacteria, and viruses, but water-quality testing of pond-sand filters installed in Bangladesh has shown that, in many cases, treatment is only partially effective, and filtered water contains high levels of coliform bacteria (2). It is critically important that when changing the source of water, the long-term risk posed by ingesting arsenic should not be replaced with an even more serious short-term risk of microbiological infection.

Boiling water for several minutes effectively kills or inactivates most protozoa, bacteria, and viruses. However, this is very expensive for rural people who use mostly twigs, crop residues, dried leaves, cowdung, and other waste biomass for cooking in clay ovens. Firewood is very costly, and widespread promotion of its use for water treatment would have a serious negative impact on the environment in Bangladesh where forest areas are greatly diminished.

Water may be purified by heat treatment without boiling, through pasteurization. Pasteurization does not sterilize water, but can reduce pathogen loads by many orders of magnitude, depending on the temperature, contact time, and heat resistance of the pathogen.

Much research has been conducted on heat inactivation of pathogens in foods, especially of bacteria. Many bacteria responsible for food poisoning are faecally transmitted and can be found in contaminated surface water. Milk is commonly pasteurized either by holding at 71.7 °C for 15 seconds, or 62.7 °C for 30 minutes (3). This achieves at least a 7-log reduction in most common bacterial pathogens. Enteric viruses and protozoa are also vulnerable to heat treatment (Table 1). The extent of destruction of pathogens increases with duration of time at elevated temperature.

**Table 1.** Thermal sensitivity of common waterborne pathogens

Pathogen	Heat sensitivity	Reference no.
Bacteria		
*Campylobacter jejuni*	Very sensitive to heat	11, 12
*Escherichia coli* O157:H7	Pasteurization is effective	13
*Enterococcus spp.*	Pasteurization is effective	12
*Salmonella typhimurium*	Standard milk pasteurization achieves >12-log reduction	12, 14
*Shigella dysenteriae*	Standard milk pasteurization is effective	15
*Vibrio cholerae*	Two minutes at 70 °C results in >7-log reduction	16
*Yersinia enterocolitica*	Holding at 71.8 °C for 18 seconds is effective	12, 17
Viruses		
Poliovirus	Completely inactivated by 30 seconds at 72 °C	18
Rotavirus	Ten minutes at 60 °C results in >7-log reduction	19
Protozoa		
*Cryptosporidium parvum*	Oocysts inactivated by heating to 72 °C for 1 minute or 45 °C for 10–20 minutes	20, 21

Thermal death kinetics are often assumed to follow a first-order rate law, although observed survival curves frequently do not match the log-linear profile predicted by this model. Tailing is frequently observed, and efforts are underway to develop improved models which more accurately reflect the actual processes at work (4).

While pasteurization is a standard practice in the food industry and is also widely used in the management of wastes (e.g. composting), it has received little attention for the treatment of drinking-water. A significant amount of work has been done on solar pasteurization of water, either through thermal treatment alone (5) or in conjunction with ultra-violet radiation (6, 7). Less attention has been given to thermal-inactivation processes. Results of one study in Kenya showed that batch pasteurization of drinking-water in households significantly improved the quality of water and reduced the incidence of diarrhoea by approximately 50% (8). This is consistent with findings of other studies which showed that household-level water-quality interventions reduce the risk of diarrhoeal diseases by 35–40% (9).

This paper presents a novel adaptation of a water-pasteurization process which has been developed in Bangladesh. Most rural communities and many urban slums in Bangladesh and the sub-continent use different types of traditional clay-ovens (*chullis*) for cooking. Since chullis are made of clay, the inside behaves like a refractory lined furnace, and internal temperature may reach more than 600 °C (10). *Chullis* are inefficient devices: more than 80% of the heat generated from combustion is wasted (10). Using a small fraction of this wasted heat, microbiologically-contaminated water may be pasteurized without incurring any extra fuel costs. Pasteurization does not improve chemical quality, so surface water containing industrial or chemical contamination should not be used for *chulli* treatment.

## MATERIALS AND METHODS

A hollow aluminium tube (12 mm internal diameter, 2.5 m long) was coiled in a helix approximately 25 cm in diameter to match the internal diameter of *chullis* commonly used in Bangladesh. The coil was then built into a traditional clay-*chulli* to create a flow-through water-treatment system.

A 20-litre plastic bucket was half-filled with washed, clean sand to serve as a rapid sand filter for the raw water, with an effluent outlet at the bottom of the bucket.

Heat-resistant plastic tubing was used for connecting the raw water bucket to the *chulli* inlet and the *chulli* outlet to a tap. While the *chulli* was used for cooking, the tap was opened to allow water to flow through the heated metal-coil. The tap was used for adjusting the flow rate, which controls the effluent water temperature. Treated water was collected in a reservoir, generally an aluminium *kolshi* (pitcher).

Effectiveness of the flow-through pasteurization treatment was assessed in the laboratory and field settings, using standard membrane-filtration analysis for total and thermotolerant coliform bacteria.

## RESULTS

### Laboratory testing

A bench model of the *chulli* system was constructed for research purposes, using a highly-contaminated pond as the source of water. It was found that, within five minutes of starting to cook a pot of rice, hot water was produced through the coil, although water in the cooking-pot was not yet warm. When the tap was adjusted to a flow rate of 500 mL per minute, corresponding to a hydraulic residence time inside the coil of roughly 45 seconds, the effluent temperature was found to be approximately 70 °C. This is also the temperature at which copious amounts of steam were emitted along with effluent water, and at which the effluent water was too hot to comfortably touched by hand. Once effluent water attained this temperature, samples were collected from the outlet tap for analysis of total and faecal coliforms in the Environmental Microbiology Laboratory of ICDDR,B using membrane filtration. The volume of water produced in each rice-cooking period was noted.

Repeated experiments showed that more than 30 litres of water could be treated during a typical cooking period of 1.5–2 hours. Since most families cook 2–3 times per day, 60–90 litres of water may be produced.

The average effluent temperature was 70–76 °C. Three sets of coliform tests of raw and treated water showed that, while the total coliform counts in the pond-water ranged from 1,750 to 560,000 cfu/100 mL, no coliform bacteria could be detected in the treated water.

### Field-testing

Working with the local NGO—Integrated Approach for Community Development (IACD)—the *chulli* water-treatment system was field-tested in three arsenic-affected upazilas: Bancharampur, Homna, and Monirampur.

In early 2004, two villages in Bancharampur and Homna were selected to pilot the *chulli* system on the basis of widespread arsenic contamination of tubewell water. The IACD staff found that most villagers were aware of the dangers posed by arsenic in drinking-water. Many families had access to rainwater systems constructed under a previous mitigation intervention, and most agreed to participate in the *chulli* pilot project. During July-September 2004, 169 *chulli* systems were installed in households in the two villages (Fig. 1). Families were instructed in their operation and were given basic information on good hygiene practices relating to water-use, such as the need to store treated water in a clean reservoir, and not to dip hands or unclean vessels into the storage reservoir. Users were instructed to take water from relatively clean sources, such as protected ponds, and to adjust the effluent tap until the water temperature was too hot to be touched.

**Fig. 1a.**
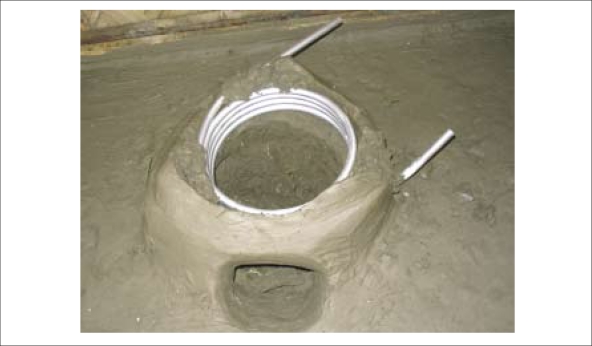
*Chulli* system being constructed

Photo credit: Richard Johnston, UNICEF

**Fig. 1b.**
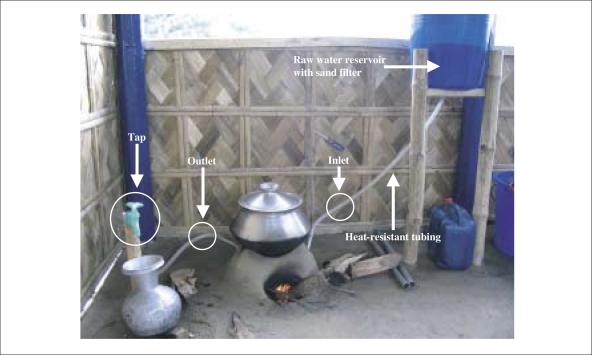
*Chulli* system components

Photo credit: Richard Johnston, UNICEF

A second phase of installation was conducted from January through March 2005, in which 447 *chullis* were installed in four additional villages in Monirampur and Bancharampur. Sources of raw water included ponds, rivers, *baors* (oxbow lakes), and rainwater. User instruction was as in Phase I.

Routine monitoring indicated that the average volume of water produced was around 90 litres for the three cooking periods usually prevalent in the villages. The average flow rate was near 500 mL per minute, and the effluent temperature was near 70 °C. Most villagers treated three batches of water a day, so that water could cool for several hours before being consumed.

**Fig. 2.**
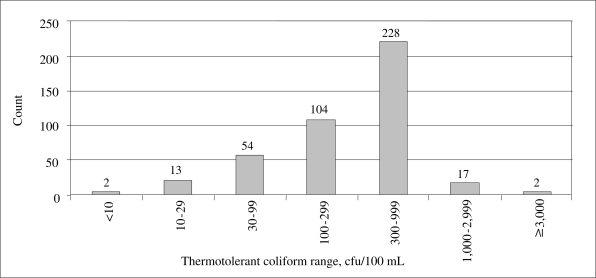
Thermotolerant bacteria counts in raw water (n=420)

In total, 420 paired samples of raw and treated water in the pre-testing and six pilot villages were analyzed for thermotolerant coliforms (Table 2) using portable membrane filtration kits (Wagtech Potatest). All the Phase I *chullis* were tested, and 20% were re-tested. As in the pre-testing, no coliforms were detected in any treated samples, and it was decided to test only half of *chullis* in Phase II. Figure 2 indicates the distribution of thermotolerant coliforms in the raw water in the pilot villages, which ranged from 9 to 4,550 cfu/100 mL (median 340). No thermotolerant coliforms were detected in any treated water samples.

**Table 2.** Field-testing of *Chulli* water-treatment system

Upazila	Villages targeted (n=6)	*Chullis* installed (n=616)	Water-quality tests made (n=420)
Pre-testing			3
Phase I			
Bancharampur	1	67	97
Homna	1	102	107
Phase II			
Bancharampur	2	177	105
Monirampur	2	270	108

In 24 Phase-II households, water that had been treated and stored overnight was tested for thermotolerant coliforms. Coliforms were found in three of these samples, at 75, 200, and 350 cfu per 100 mL. In all three cases, water had been stored with a cover for 2–4 days.

The *chulli* system was socially acceptable. Most villagers know that hot food and boiled water are free from dangers of diarrhoea. The idea that hot water can be obtained so easily without any extra cost was very new to them and motivated people to quickly accept the technology. The simple technology, occupying almost no extra space in the household, fascinated people. Many families were astonished by the large amount of water produced and even complained that the system produced more treated water than they could conveniently store. Some families shared their water with nearby neighbours who did not have access to arsenic-free water.

In fall 2004, the two Phase-I project villages were severely flooded, and many villagers took shelter in a nearby school with a second story. IACD workers constructed two portable *chullis* for use at the flood shelter. Almost 60 families were served with potable hot water and meals for nearly 20 days. After the floods receded, nearly all the clay-*chullis* constructed in the two villages had been washed away and needed to be re-built. However, the aluminium coils were undamaged, and women were able to re-build their *chullis* by themselves.

## DISCUSSION

Laboratory experiments and field-testing have established the technical viability of the *chulli* water-treatment system for effective removal of coliform bacteria from moderately-contaminated surface water through pasteurization. Result of a literature review also indicated that most common waterborne pathogens, including rotavirus, the leading viral cause of diarrhoeal disease, and *Cryptosporidium parvum* oocyts, are vulnerable to treatment of pasteurization. In conventional pasteurization of food, elevated temperatures are only held for a few seconds or minutes. Water treated in the *chulli* system remains at elevated temperatures for much longer as it cools, affording additional time for inactivation of pathogens. Although the current study measured only coliform indicator species, it is likely that most common enteric pathogens (Table 1) will be inactivated through *chulli* treatment.

The system is simple and inexpensive (costing about US$ 6) and requires no operating and maintenance expenses. A household can treat more than enough water for domestic use, with no additional time or fuel. Users in the pilot areas liked the system and appreciated the availability of hot, pasteurized water in the household.

Where groundwater is contaminated with arsenic (or fluoride), *chulli* treatment may provide a good alternative, if acceptable sources of surface water are available. However, the *chulli* water treatment has the potential for broader application. Millions of people throughout the developing world are using microbiologically-contaminated drinking-water and cooking-water, which could be made safe through this flow-through pasteurization process. The *chulli* technology presents a new opportunity for low-cost, effective treatment of water at the household level.

Portable *chulli* systems could also be used in public settings where cooking is done and hygiene standards are not high, such as launches, steamers, tea shops, and restaurants. The concept could even be extended to industrial settings, such as rice-making plants and brick kilns, where large amounts of heat are lost to the environment.

Unlike chemical disinfection methods, pasteurization provides no residual protection against re-contamination. Results of numerous studies have shown that, when unchlorinated water is stored in rural households, coliform bacteria counts can rapidly rise, even if quality of water is good at the source of water. Stored water can be contaminated through collection in unclean vessels, contact with hands and unclean materials during transport, and dipping of unclean vessels (cups, ladles, etc.) into the storage reservoir in the home. In the *chulli* system, the high temperature of collected water will help kill any pathogens present in unclean storage containers, affording some protection against secondary contamination. However, good water-hygiene practices, such as keeping the storage reservoir covered and not dipping vessels into the reservoir, are critical to prevent secondary contamination of treated water and should always be promoted during the training of users of the new *chulli* system.

## ACKNOWLEDGEMENTS

The authors acknowledge the financial help of UNICEF for the pilot project, the support of Mr. S.M. Ihtishamul Huq (Department of Public Health Engineering), and the keen interest and hard work of the Integrated Approach for Community Development staff.
